# *QuickStats:* Percentage of Newborns Breastfed Between Birth and Discharge* from Hospital, by Maternal Age — National Vital Statistics System, 49 States^†^ and the District of Columbia, 2021 and 2022

**DOI:** 10.15585/mmwr.mm7304a6

**Published:** 2024-02-01

**Figure:**
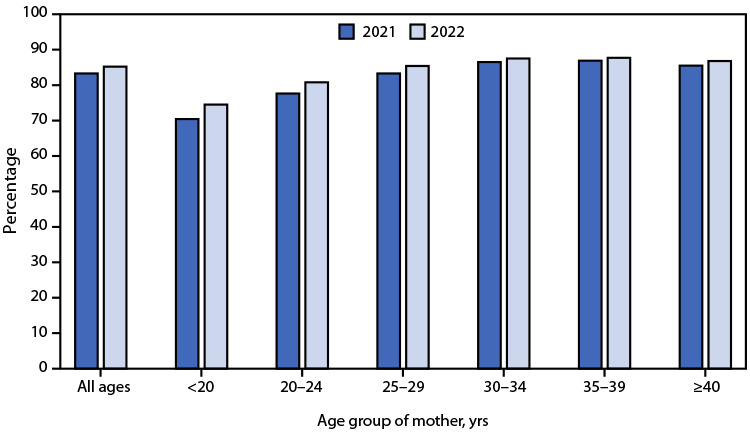
Among 49 states and the District of Columbia, the percentage of newborns breastfed between birth and discharge from the hospital increased from 83.3% in 2021 to 85.2% in 2022. Increases were observed for each maternal age group; the largest increases occurred among younger maternal age groups (70.4% to 74.5% among mothers aged <20 years and 77.6% to 80.8% among mothers aged 20–24 years). Despite the recent increases in initiation of breastfeeding at birth among younger mothers, older mothers were still more likely to breastfeed their newborns (86.8% of those aged ≥40 years versus 74.5% of mothers aged <20 years).

**Source:** National Center for Health Statistics, National Vital Statistics System, Natality Data, 2021 and 2022. https://www.cdc.gov/nchs/nvss/births.htm

